# Eating Problems and Overlap with ADHD and Autism Spectrum Disorders in a Nationwide Twin Study of 9- and 12-Year-Old Children

**DOI:** 10.1155/2013/315429

**Published:** 2013-04-15

**Authors:** Maria Råstam, Jakob Täljemark, Armin Tajnia, Sebastian Lundström, Peik Gustafsson, Paul Lichtenstein, Christopher Gillberg, Henrik Anckarsäter, Nóra Kerekes

**Affiliations:** ^1^Department of Clinical Sciences, Lund, Child and Adolescent Psychiatry, Lund University, Sofiavägen 2D, SE-22241 Lund, Sweden; ^2^Centre for Ethics, Law and Mental Health (CELAM), University of Gothenburg, Wallinsgatan 8, SE-43141 Mölndal, Sweden; ^3^Swedish Prison and Probation Service, R&D Unit, Gothenburg, Wallinsgatan 8, SE-43141 Mölndal, Sweden; ^4^Gillberg Neuropsychiatry Centre, Institution of Neuroscience and Physiology, University of Gothenburg, Kungsgatan 12, SE-41119 Göteborg, Sweden; ^5^Department of Medical Epidemiology and Biostatistics, Karolinska Institutet, Nobels Väg 17, SE-17165 Solna, Sweden

## Abstract

*Aim*. To establish the prevalence of restrictive eating problems, the overlap and association with attention-deficit/hyperactivity disorder (ADHD), and autism spectrum disorders (ASD) and to estimate the heritability of eating problems in a general population sample of twins aged 9 and 12. *Methods*. Parents of all Swedish 9- and 12-year-old twin pairs born between 1993 and 1998 (*n* = 12,366) were interviewed regarding symptoms of ADHD, ASD, and eating problems (EAT-P). Intraclass correlations and structural equation modelling were used for evaluating the influence of genetic and environmental factors. Cross-twin, cross-trait correlations were used to indicate a possible overlap between conditions. *Results*. The prevalence of eating problems was 0.6% in the study population and was significantly higher in children with ADHD and/or ASD. Among children with eating problems, 40% were screened positive for ADHD and/or ASD. Social interaction problems were strongly associated with EAT-P in girls, and impulsivity and activity problems with EAT-P in boys. The cross-twin, cross-trait correlations suggested low correlations between EAT-P and ADHD or EAT-P and ASD. Genetic effects accounted for 44% of the variation in liability for eating problems. *Conclusions*. In the group with eating problems, there was a clear overrepresentation of individuals with ADHD and/or ASD symptoms.

## 1. Introduction

In typically developing younger children, the prevalence of the clinical eating disorders is low [1, 2], with one large-scale study reporting a prevalence of 0.15% for DSM-IV eating disorders in 11- to 12-year olds [3]. However, some degree of milder eating problems is relatively common, affecting from 20 to 40 percent of children [1]. Selective eating or picky or faddy eating is a transient problem in over 10% of all toddlers [2]. A recent surveillance study [4] based on close to 2500 Canadian paediatricians' reports on “any disordered eating behavior sufficient to cause a disruption, weight gain, or actual loss of weight” found 161 children from 5 to 12 years of age. The highest incidence, 9.4 cases per 100 000 person-years, was found in girls aged from 10 to 12 years (1.3 for boys). 

Dieting as a general, non-specific risk factor increases the risk of developing an eating disorder by about five times [5]. It has been suggested that subclinical variants of eating disorders start at an earlier age now than was the case in the twentieth century and that the prevalence of early dieting/restrictive eating is increasing [6]. While eating problems in childhood may be a risk factor for the development of eating disorders in adolescence and young adulthood [7–9], a comprehensive review on risk factors for eating disorders stressed a need for larger-scale studies [10]. 

Children with early symptomatic neuropsychiatric disorders have been found to have high frequencies of feeding/eating problems [11, 12] compared to children without such disorders, but there have been few, if any, large-scale studies in the general population investigating this problem [1].

As far as we are aware, there are few studies on heritability in prepubertal eating problems/eating disorders. In one study of over 5000 twins aged from 8 to 11 years, parent-reported food neophobia was highly heritable explaining 78% of the variance while 22% was explained by nonshared environmental factors [13].

The present paper assesses the rate of eating problems in a large young cohort of twins from the general population. Results are broken down by gender, genetic background factors, and by validated screening diagnoses of ADHD and ASD. In addition, we examined which facets of ADHD and ASD that had the strongest associations with eating problems.

## 2. Methods

### 2.1. Subjects

The Child and Adolescent Twin Study in Sweden (CATSS) is an ongoing longitudinal twin study targeting all twins born in Sweden since July 1, 1992. Since 2004, parents of twins are interviewed regarding their children's somatic and mental health and social environments in connection with the children's 9th or 12th birthdays (CATSS-9/12), with an overall response rate of 80% of all families contacted [14].

Parental information on 12,496 children from the birth cohorts between 1993 and 1998 of CATSS was used for analysis. In the present study, 130 individuals were excluded because they had known severe brain damage or known chromosomal aberrations, leaving data on 12,366 individuals (6331 boys and 5996 girls). In a further 62 cases there were missing items on key variables. Therefore in analyses including all key variables 12,304 children (3023 boys and 2852 girls aged 9 and 3296 boys and 3133 girls aged 12) were included. 

### 2.2. Measures

#### 2.2.1. The A-TAC Inventory

All twins participating in the study were screened for possible neurodevelopmental problems using a specially developed inventory, the Autism-Tics, ADHD, and other Comorbidities (A-TAC) inventory, including a previously used algorithm for eating problems and validated algorithms for ADHD and ASD [15]. 

The A-TAC inventory includes questions to investigate child psychiatric problems based on criteria stated in the *Diagnostic and Statistical Manual of Mental Disorders, *4th edition [16]. The A-TAC was designed for use in large-scale epidemiological research as an easy-to-administer, dimensional, and comprehensive parental interview that can be carried out by lay interviewers over the phone [15, 17]. The instrument is freely available as additional web material to the second validation study [15]. Items are organized into modules (e.g., Concentration/Attention and Impulsiveness/Activity form the ADHD domain, and Language, Social interaction, and Flexibility form the ASD domain). Modules are assessed without diagnostic hierarchies or exclusion criteria.

#### 2.2.2. Questions and Scoring

In two validation studies [15, 17] lower cutoffs for screening purpose and higher cutoffs for use as clinical proxies have been defined for both the ADHD and the ASD scales. In the present study we have used the lower cutoffs for identifying children screening positive for ADHD (scores ≥6; sensitivity, 0.98; specificity, 0.81) and/or ASD (scores ≥4.5; sensitivity, 0.96; specificity, 0.88) [15]. 

Modules used in the present study were Concentration and Attention, Impulsiveness and Activity, Language, Social interaction, Flexibility, and Feeding/Eating. Each module starts with a reminder that the questions refer to a lifetime perspective, in comparison to peers, and that the questions addressing specific symptoms or characteristics may be answered by the response categories “no” (score 0), “yes, to some extent” (score 0.5), and “yes” (score 1.0). As alternatives, “do not know” or “do not wish to answer” are given, both of which are coded as “missing.” 

The Eating module screens for restrictive eating problems. Eating problems “(EAT-P)” was defined here as scoring ≥1.5 on the collapsed score for the two key questions of the Eating module [15]. These questions are (1) has s/he ever failed to gain enough weight for more than a year? (2) Has s/he seemed fearful of gaining weight or growing fat?

### 2.3. Statistical Analyses

#### 2.3.1. Association Analyses

To investigate the association between the different facets of ADHD: (1) Concentration/Attention and (2) Hyperactivity/Impulsiveness, and ASD: (1) Language, (2) Social interaction, and (3) Flexibility- and EAT-P we used a binary logistic regression response model with data on 12,366 children. To account for the dependency within twin pairs a generalized estimation equation (GEE) model was fitted to the data. All variables were inserted as continuous covariates, except age. In a first step all factors were assessed in a univariate model, and, in a second step, a multivariate model was created that only included significant associations from the univariate model.

#### 2.3.2. Twin Statistics

Twin methodology is based on the comparison of monozygotic and dizygotic twin pairs. Monozygotic twins share all their genes, while dizygotic twins, on average, share 50% of their segregating alleles. This makes it possible to disentangle genetic from environmental components of a trait or condition. In twin methodology, etiological factors are partitioned into genetic (A) factors, shared environmental (C) factors (factors that make the twins more similar), and nonshared environmental factors (E) (factors that make twins dissimilar). Intraclass correlations and standard continuous univariate heritability models were calculated in Mx [18]. We did not attempt to reduce the models since that can lead to biases in the observed estimates [19]. Cross-twin, cross-trait correlations (the continuous score of trait 1 in twin 1 is correlated with the continuous score of trait 2 in twin 2) were calculated using the PROC CORR procedure in SAS 9.3. Cross-twin, cross-trait correlations are used to indicate if common genetic and environmental effects over two traits existed. If the correlation is higher for monozygotic twins than for dizygotic twins, then common genetic effects influencing both traits are implicated. As the cross-twin cross traits correlations were quite similar for monozygotic and dizygotic twins, we did not go on to attempt bivariate model fitting. 

Zygosity was determined in over 90% of the twins with a panel of 48 single nucleotide polymorphisms or, for those twins where DNA samples were missing, with the help of validated algorithms [20].

## 3. Results

### 3.1. Prevalence of EAT-P, ADHD, and ASD

Of the 12,304 children included in the present study, 903 were screened positive for ADHD only (scoring ≥6.0 in the ADHD and <4.5 in the ASD blocks), 89 were screen positive for ASD only (scoring ≥4.5 in the ASD and <6 in the ADHD blocks), and 288 children were screened positive for both ASD and ADHD. The rest of the children (*n* = 11, 024) constituted the comparison group. 

The prevalence of EAT-P was low (*n* = 72; 0.6%) of the total population of 12,304 children aged 9 and 12, with a close-to-equal distribution between ages, and a predominance of girls (30 boys and 42 girls). In the comparison group of 11,024 children with no ADHD/ASD, there were 43 children with EAT-P (0.4%; boys 0.2%, girls 0.6%) (Table 1). In the group of children with ADHD and/or ASD (*n* = 1280) there were 29 children with EAT-P (2%; boys 2%, girls 3%). The prevalence of EAT-P was significantly higher in the group of children with ADHD and/or ASD compared to the group of children with no ADHD and no ASD (*P* < 0.001). The highest prevalence of EAT-P was seen in children scoring positive for both ADHD and ASD (5.6%; boys 4.2%, girls 9.7%).

### 3.2. Prevalence of ADHD and ASD in Children with and without EAT-P

**Figure 1:**
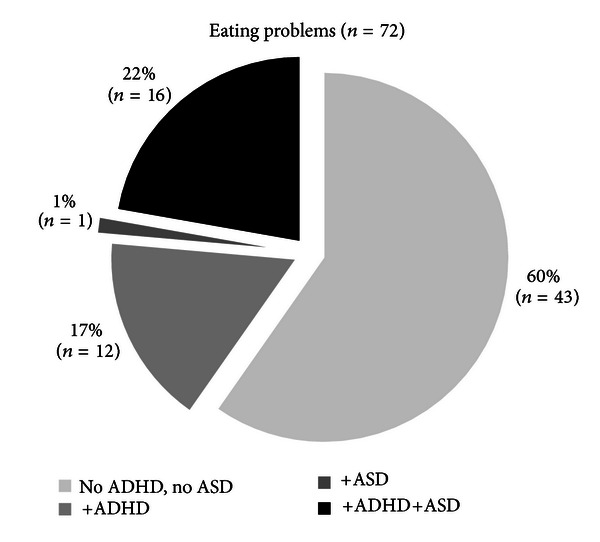
Prevalence of ADHD and/or ASD in the children with EAT-P. EAT-P: eating problems; ADHD: attention deficit hyperactivity disorder; ASD: autism spectrum disorder.

Forty percent of all children who were reported to have EAT-P and 10% of those without EAT-P were screen positive for ADHD and/or ASD, as shown in Figure 1.

### 3.3. Associations with Subdomains of ADHD and ASD

Table 2 summarizes the association in measures of odds ratios (ORs) between EAT-P and age and between modules of ADHD and ASD, separately for both genders. Both ADHD modules (Concentration/Attention, and Impulsiveness/Activity) and all three ASD modules (Language, Social interaction, and Flexibility) were significantly associated with EAT-P in both genders in the univariate models. For example, for each new Concentration/Attention symptom the risk of eating problems increased with 36% in boys and with 33% in girls (OR = 1.36/1.33; CI = 1.21–1.53/1.16–1.52, resp.). When fitting all the significant variables of EAT-P into a multivariate model, only three variables were significantly associated with EAT-P. These were Social interaction problems (OR = 1.95, *P* < 0.005) for girls, and for boys Impulsiveness/Activity problems (OR = 1.41, *P* < 0.001), and age 9 years compared to 12 years (OR = 0.37, *P* < 0.05). 

### 3.4. Heritability

Intraclass correlations were at least twice as strong in monozygotic pairs as in dizygotic same-sex pairs, both generally and in each gender separately (Table 3). Genetic effects (heritability) accounted for 44% of the variance in EAT-P. There was no indication of shared environmental effects. The remaining variance was due to nonshared environmental effects. The phenotypic correlations did not exceed 0.23, and the cross twin, cross trait correlations suggested low correlations (<0.20) between EAT-P and ADHD or EAT-P and ASD, which did not differ substantially between monozygotic and dizygotic twins. 

## 4. Discussion

EAT-P in the present study was defined by parent-reported weight stop/loss combined with fear of gaining weight in the child, and the main findings were as follows.

- The prevalence of EAT-P was low (under one per cent) in these cohorts of 9- and 12-year olds.
- The prevalence of EAT-P was significantly higher in children who also screened positive for ADHD and/or ASD, with the highest prevalence of EAT-P, almost ten percent, reported for girls who screened positive for both ADHD and ASD.
- Social interaction problems were strongly associated with EAT-P in girls, and impulsivity and activity problems were strongly associated with EAT-P in boys.
- In childhood, eating problems seemed to be in equal parts accounted for by genetic and nonshared environmental background factors.

Based on earlier published reports, the low prevalence of restrictive eating in the age cohorts in the present study was to be expected [2, 5]. However, as far as we are aware there have been few studies on the general population of 9 to 12-year olds. Furthermore, few existing reports have looked specifically at the critical prepubertal years, critical if the purpose is to examine early onset of restrictive eating [21]. The expected overrepresentation of girls could be expected from all previous epidemiological studies. In boys there was a significant increase in EAT-P with age. However, earlier literature also gives the expectation of an increasing prevalence of restrictive eating in 12-year old girls compared to 9-year olds [22] which was not substantiated in this study. 

The prevalence of EAT-P, at least as defined in this study, was relatively low compared to other developmental problems [15]. Similar to some previous studies [23, 24], in the present study children screening positive for ADHD and/or ASD had an increased risk of eating problems causing weight loss. In the children with such eating problems there was a clear overrepresentation of individuals with ADHD and/or ASD. Concerning this finding there are few studies except a study in UK of a nonclinical sample of 132 schoolchildren with similar results [25]. Eating disorders are now considered to be neurodevelopmental disorders [26], and a link with childhood obsessive-compulsive personality traits [27], and even with ASD, has been suggested [28, 29]. The neurodevelopmental disorders should be considered in children with eating disorders, especially in girls where mild forms of ADHD and ASD tend to be overlooked [30]. 

Gender specific differences could be seen concerning neurodevelopmental problems associated with EAT-P. Hyperactivity and impaired social interaction showed strong and significant association to EAT-P for both genders. As far as we know it is a new finding for prepubertal eating symptoms, but it would seem to be in agreement with earlier literature on adolescent onset eating disorders [31–33]. However, in the multivariate analysis, the strongest association of EAT-P for girls was problems with social interaction, and in boys the strongest association was with hyperactivity and impulsivity. These gender differences seem to be in agreement with some reports of excessive exercise as more common in male than in female eating disorders [34]. 

Eating problems in 9 to 12-year olds appear, similar to later in adolescence [29, 35], to have an equally large genetic and non-shared environmental background. However, the scant literature of boys and men with eating problems/eating disorders does not allow any comparing with earlier findings. The very similar cross-twin cross-trait correlations together with the low phenotypic correlation suggested that the small part of variance that is shared between the conditions is mainly due to shared environmental factors. Future studies should investigate if this association is similar above the diagnostic threshold. A review [22] stressed the complexity of influences on eating behaviours and weight as parents provide both the genetic predispositions and the environment (the food and the attitudes to food) in which these predispositions are expressed. Maternal food intake strongly correlates with child food intake [30]. The clinical implications of the interplay between environmental and genetic risk factors for eating disorders have been comprehensively described in a recent review [36].

The strengths and limitations of the study should be taken into account in interpreting findings. The population-based nature of the study sample is an important strength. An obvious limitation is that the information regarding symptoms and behaviour consisted of parent ratings in a telephone interview. The A-TAC inventory has a proven excellent ability to distinguish children with neurodevelopmental problems from children with no such problems [14], but it has not been validated for the assessment of eating problems which suggests that the results should be interpreted with caution. The focus of this study is on restrictive eating, and questions on bingeing and obesity have not been included in the analyses in the present study. The study was cross-sectional and could not say anything about causality.

The clinical implication of this study is that neurodevelopmental disorders should be considered in children with disordered eating, and, conversely, that eating problems/disorders should be considered in children with ADHD and/or ASD. Interventions must be matched to the patient, and only if neurodevelopmental aspects are considered in each individual case, one can expect results.

## Figures and Tables

**Table 1:** Prevalence of EAT-P.

Groups	Total study group^a^ *n* (boys + girls)	EAT-P *n* (boys + girls)	% within the group (boys + girls)
ADHD only	903	12	1.33
	(601 + 302)	(8 + 4)	(1.33 + 1.32)
ASD only	89	1	1.12
	(61 + 28)	(1 + 0)	(1.64 + 0.00)
ADHD + ASD	288	16	5.56
	(216 + 72)	(9 + 7)	(4.17 + 9.72)
Comparison (no ADHD, no ASD)	11024	43	0.39
	(5441 + 5583)	(12 + 31)	(0.22 + 0.56)
Study population (boys + girls)	12304^a^	72	0.59
	(6319 + 5985)	(30 + 42)	(0.47 + 0.70)

^a^Excluding 62 individuals for whom items were missing on the response variables yielded 12,304 individuals for prevalence analyses.

EAT-P: eating problems; ADHD: attention deficit hyperactivity disorder; ASD: autism spectrum disorder.

**Table 2:** Measuring associations between EAT-P and subdomains of ADHD and ASD, for boys and girls separately by GEE models.

Factors/covariates	Crude measures				Univariate model		Multivariate model^a^	
	*n*	Min–max	*M*	SD	OR	95% CI	OR	95% CI
Boys					EAT-P (Prevalence 0.5%)			

Age 9	3029	—	—	—	0.40*	0.18–0.89	0.37*	0.16–0.84
Age 12 (reference group)	3302	—	—	—	1	—	1	—
Concentration/attention problems	6325	0–9	1.26	1.90	1.36***	1.21–1.53	0.98	0.76–1.26
Impulsiveness/activity problems	6326	0–10	1.09	1.78	1.46***	1.34–1.60	1.41***	1.18–1.69
Language problems	6326	0–6	0.31	0.67	1.97***	1.67–2.33	1.33	0.85–2.08
Social interaction problems	6318	0–6	0.30	0.69	1.76***	1.48–2.09	0.70	0.44–1.12
Flexibility problems	6329	0–5	0.31	0.68	2.07***	1.68–2.55	1.42	0.86–2.37

Girls					EAT-P (prevalence 0.7%)			

Age 9	2858	—	—	—	0.65	0.34–1.28		—
Age 12 (reference group)	3138	—	—	—	1	—		—
Concentration/attention problems	5992	0–9	0.74	1.43	1.33***	1.16–1.52	0.94	0.74–1.18
Impulsiveness/activity problems	5988	0–10	0.70	1.37	1.41***	1.26–1.58	1.18	0.94–1.47
Language problems	5993	0–6	0.19	0.47	2.32***	1.82–2.97	1.27	0.77–2.11
Social interaction problems	5976	0–6	0.20	0.50	2.45***	2.02–2.98	1.95**	1.22–3.10
Flexibility problems	5995	0–5	0.17	0.46	2.10***	1.57–2.81	0.89	0.52–1.51

*N* = 12,366, boys *n* = 6331, Girls *n* = 5996; EAT-P: eating problems; ^a^with significant variables of the univariate models; *P < 0.05; **P = 0.005; ***P < 0.001.

**Table 3:** Intraclass correlations, heritability estimates, and cross-twin cross-trait correlations for the collapsed sample and by gender.

	Intraclass correlations (95% CI:s)		Heritability estimates (95% CI:s)			Cross-twin, cross-trait correlations (95% CI:s)				Phenotypic correlations (95% CI:s)	
EAT-P	MZ	DZ ss	A	C	E	ADHD		ASD		ADHD	ASD
						MZ	DZ ss	MZ	DZ ss		
ALL	0.42	0.18	0.44	0.00	0.56	0.16	0.13	0.15	0.11	0.15	0.20
	(0.39–0.45)	(0.15–0.21)	(0.40–0.48)	(0.00–0.02)	(0.53–0.60)	(0.11–0.21)	(0.09–0.17)	(0.10–0.19)	(0.07–0.15)	(0.13–0.18)	(0.18–0.22)
Boys	0.34	0.10	0.32	0.00	0.68	0.19	0.15	0.17	0.14	0.18	0.23
	(0.30–0.38)	(0.06–0.14)	(0.25–0.38)	(0.00–0.05)	(0.62–0.73)	(0.12–0.25)	(0.09–0.21)	(0.10–0.24)	(0.08–0.20)	(0.15–0.21)	(0.20–0.26)
Girls	0.48	0.23	0.53	0.00	0.47	0.16	0.14	0.17	0.09	0.16	0.18
	(45–0.52)	(0.18–0.27)	(0.43–0.52)	(0.00–0.04)	(0.43–0.52)	(0.09–0.22)	(0.08–0.20)	(0.10–0.23)	(0.02–0.15)	(0.12–0.19)	(0.15–0.21)

EAT-P: eating problems, MZ: monozygotic, DZ-ss: dizygotic same sex, CI:s: confidence intervals.

A: genetic factors, C: shared environmental factors, and E: nonshared environmental factors.

Pairs where information was eligible from both twins were included in the analyses, giving a total of 1620 MZ boys, 1694; DZ girls, 2310 DZ-ss boys 2310, and 1944 DZ-ss girls.
